# Associations of low-density lipoprotein cholesterol and hemoglobin A1C with cardiovascular events and mortality in breast and prostate cancer patients

**DOI:** 10.1016/j.ijcrp.2025.200468

**Published:** 2025-07-11

**Authors:** Yen-Chou Chen, Jhih-Yuan Lu, Chun-Yao Huang, Yu-Hsuan Joni Shao

**Affiliations:** a Division of Cardiology and Cardiovascular Research Center, Taipei Medical University Hospital, Taipei, Taiwan; b Taipei Heart Institute, Taipei Medical University, Taipei, Taiwan; c Office of Data Science, Health Data Analytics and Statistics Center, Taipei Medical University, Taipei, Taiwan; d Department of Biomedical Sciences and Engineering, National Central University, Taoyuan, Taiwan; e Graduate Institute of Biomedical Informatics, College of Medical Science and Technology, Taipei Medical University, Taipei, Taiwan; f Clinical Big Data Research Center, Taipei Medical University Hospital, Taipei, Taiwan

**Keywords:** Cardio-oncology, Low-density protein cholesterol, Hemoglobin A1C, Cancer survivors, Cardiovascular disease

## Abstract

**Background:**

Cardiovascular disease is a major non-cancer cause of morbidity in cancer patients. Low-density lipoprotein cholesterol (LDL) and hemoglobin A1C (HbA1C) are shared risk factors for cancer and cardiovascular disease. However, optimal management for these factors in cancer patients remains unclear.

**Methods:**

This retrospective cohort study investigated associations between LDL and HbA1C levels with major cardiovascular events, all-cause mortality, and cancer recurrence in patients with breast and prostate cancer. The analysis included 832 breast cancer and 593 prostate cancer patients from the Taipei Medical University Clinical Research Database (2011–2020), using Cox proportional hazard models with time-dependent covariates. The findings were validated using the TriNetX research network, using a propensity score matching method.

**Results:**

Elevated LDL levels (≥130 mg/dL) were associated with a higher risk of major cardiovascular events, particularly in prostate cancer patients. A U-shaped association was observed between LDL levels and all-cause mortality, with the lowest risk in the 100–129 mg/dL range (propensity score matching risk ratios for all-cause mortality with LDL ≥130 mg/dL: 1.05 [95 % confidence interval 1.02–1.09] for breast cancer and 1.08 [95 % confidence interval 1.04–1.12] for prostate cancer). Elevated levels of HbA1C (≥6 %) were also associated with increased risks of cardiovascular events, with a potential U-shaped association with mortality.

**Conclusion:**

Higher levels of LDL and HbA1C are associated with increased risks of cardiovascular events and all-cause mortality in breast and prostate cancer patients, supporting current cardio-oncologic guidelines in cancer survivors.

## Highlights

• Cardiovascular disease (CVD) is a major non-cancer cause of morbidity and mortality in cancer patients.
• Low-density lipoprotein cholesterol (LDL) and hemoglobin A1C (HbA1C) are shared risk factors for cancer and CVD.
• Higher levels of LDL and A1C were associated with increased risks of CVD and mortality in cancer patients.
• Managing LDL and A1C in cancer survivors based on contemporary guidelines may reduce the risks of death and CVD.

## Graphical abstract

**Graphic Abstract.** Low-density protein cholesterol ≥130 mg/dL & hemoglobulin A1C ≥ 6 % were associated with higher risks of cardiovascular risk and all-cause mortality. A U-shaped association of Low-density protein cholesterol with all-cause mortality was observed (the lowest risk in 100–129 mg/dL). CI = confidence interval; HbA1C = hemoglobulin A1C; LDL = low-density protein cholesterol.

**Figure:**
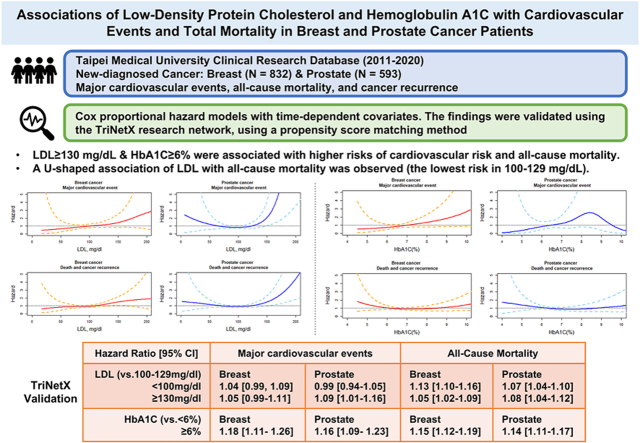

## 1 Introduction

Cardiovascular disease (CVD) is a leading non-cancer cause of morbidity and mortality in cancer patients [1]. Although clinical guidelines for managing cardiovascular risks—such as diabetes mellitus and dyslipidemia—are well established [2,3], cancer patients are frequently excluded from major cardiovascular trials [4]. Therefore, current cardio-oncology recommendations are mostly extrapolated from studies in the general population. The optimal strategies for cardiovascular risk management in cancer patients remain unclear.

Low-density lipoprotein cholesterol (LDL) and hemoglobin A1C (HbA1C) levels are shared risk factors for cancer and CVD [5,6]. Elevated LDL level may be associated with higher cardiovascular risks in both general population and cancer survivors [[7], [8], [9], [10]]. Notably, prior studies in the general population showed a U-shaped association between LDL levels and all-cause or cancer-related mortality [7,8], raising the uncertainty about optimal cholesterol strategies in cancer patients. Similarly, elevated HbA1C increases risks of all-cause mortality and cardiovascular events, even in non-diabetic populations [[11], [12], [13]]. In cancer patients, elevated HbA1C levels may increase cardiovascular risks [14,15] and worsen cancer prognosis [[16], [17], [18]]. However, most existing evidence came from studies of the general population or heterogenous cancer types, limiting its generalizability and applicability for specific cancer populations.

This study aimed to address the associations of LDL and HbA1C levels with major cardiovascular events (MACE), all-cause mortality, and cancer recurrence in patients with breast and prostate cancer, utilizing the Taipei Medical University Clinical Research Database (TMUCRD) and the TriNetX network research database.

## 2 Method

### 2.1 Study sample

This retrospective cohort study used the TMUCRD and the TriNetX research network to evaluate and validate the associations of LDL and HbA1C with cancer-related and cardiovascular outcomes. The TMUCRD contains electronic medical records of roughly 4.3 million patients between 1998 and 2022 at three affiliated hospitals of Taipei Medical University: Taipei Medical University Hospital, Taipei Wan-Fang Hospital, and Shuang-Ho hospital, Ministry of Health and Welfare [19]. The TriNetX research network comprises data from the electronic health records of approximately 140 million patients from 112 health care organizations by Feb 7th, 2024, including diagnoses, procedures, medications, and laboratory results. The TriNetX platform only contains retrospectively deidentified data per the deidentification standard defined in section §164.514(a) of the Health Insurance Portability and Accountability Act Privacy Rule [20]. Both databases are based on the International Classification of Diseases for disease coding (Supplemental Table 1). This study only used deidentified and delinked information and was exempt from informed consents. This study was approved by the Institutional Review Board of Taipei Medical University (TMU-JIRB No. N202011006), with a waiver of informed consent.

We identified 12,180 females with newly diagnosed breast cancer and 3882 males with newly diagnosed prostate cancer, all aged ≥20 years, from the TMUCRD between June 1st, 2011, and June 30th, 2020. Patients were excluded if they had fewer than two measurements of serum LDL or HbA1c during the follow-up period (Supplemental Figs. 1 and 2). Average values of LDL or HbA1c were calculated using all available measurements prior to cancer diagnosis. Patients were stratified to assess the impacts of serum LDL and HbA1c on the MACE and cancer-related outcome [2,3]. For LDL, patients were stratified into three groups: ≥130 mg/dl, 100–129 mg/dl, and <100 mg/dl. For HbA1c, patients were stratified into ≥6.0 % or <6.0 %. The groups with LDL 100–129 mg/dl and HbA1c<6.0 % were used as reference groups.

### 2.2 Measures

This study evaluated the first incidents of MACE (a composite hospitalization outcome for myocardial infarction, ischemic stroke, and heart failure), cancer recurrence, and all-cause mortality after cancer diagnosis. These outcomes were identified with the first inpatient diagnosis after the cancer diagnosis. Due to the small number of events, a secondary outcome assessed the composite outcome of cancer recurrence and all-cause mortality. Patients were followed from the date of cancer diagnosis until the occurrence of study outcomes, death, or the end of follow-up (June 30th, 2020), whichever came first.

Clinical covariates included demographics, cancer stages, CV comorbidities, and relevant therapies [21]. The covariates were screened between June 1st, 2011, and the date of cancer diagnosis, based on at least one inpatient claim or three outpatient claims. Cancer therapy was identified between the date of cancer diagnosis and the occurrence of study outcomes. Use of cardiovascular medications was defined by the presence of at least one in-hospital or outpatient prescription prior to cancer diagnosis.

### 2.3 Statistical analysis

Baseline characteristics were compared across LDL and HbA1c groups. Categorical variables were analyzed using the Chi-square test or Fisher's exact test. The Shapiro-Wilk test was used to assess the normality of continuous variables. For normal distribution data, Student's *t*-test and one-way ANOVA test were used. For data without normal distribution, the Kruskal-Wallis test was used.

Given the potential fluctuation in LDL or HbA1C over time, Cox proportional hazard models with time-dependent covariates [22] were used to estimate hazard ratios (HR) with 95 % confidence interval (CI), for MACE, cancer recurrence or all-cause mortality. Three models were built: Model 1 was unadjusted; Model 2 was adjusted for age; Model 3 was adjusted for age, cardiovascular comorbidities, chronic lung disease, chronic kidney disease, cancer characteristics, and relevant therapies. Only covariates without missing data were included for model adjustment. The proportional hazard assumption was examined using Schoenfeld residuals. Due to limited numbers of patients and events from the TMUCRD, subgroup analysis by specific events or cancer stages were not performed. All analyses were conducted using SAS version 9.4 (SAS Institute), with statistical significance defined as a two-sided P value < 0.05.

To validate the findings of the TMUCRD, we identified 670,458 females with newly diagnosed breast cancer and 512,016 males with newly diagnosed prostate cancer, all aged ≥20 years, from the TriNetX network database between June 1st, 2011, and June 30th, 2020. Patients were included only if they had at least one measurement of LDL or HbA1c within one year prior to cancer diagnosis. Using the built-in TriNetX function, propensity score (PS) matching was performed in a 1:1 or 1:1:1 ratios by greedy closest neighbor matching without replacement [23,24]. Model 1 was matched for age at cancer diagnosis. Model 2 was matched for age at cancer diagnosis, cardiovascular comorbidities, chronic lung disease, chronic kidney disease, and relevant cancer therapy. Standardized mean differences <0.1 indicated a negligible difference between matched groups. Cox proportional hazard models were used to assess the associations of MACE, cancer recurrence, or all-cause mortality with different levels of LDL or HbA1C, respectively. Kaplan-Meier curves were applied for the survival probability, with the statistical significance of the p-value <0.05.

## 3 Results

We selected 832 females with breast cancer and 593 males with prostate cancer, each with ≥2 LDL measurements (Supplemental Tables 2 and 3). Patients with LDL<100 mg/dl were older (median age 67.2 years in breast cancer; 76.8 years in prostate cancer) and had more cardiovascular comorbidities (values reported as LDL <100, 100–129, and ≥130 mg/dL). In both cancers, the LDL <100 mg/dL group had higher rates of heart failure (breast: 14.1 % vs. 8.4 % vs. 6.1 %; prostate: 19.0 % vs. 13.5 % vs. 5.1 %), ischemic stroke (breast: 12.5 % vs. 8.2 % vs. 4.0 %; prostate: 24.9 % vs. 17.6 % vs. 6.8 %), hypertension (breast: 71.8 % vs. 63.6 % vs. 44.4 %; prostate: 80.3 % vs. 75.1 % vs. 49.2 %), diabetes mellitus (breast: 52.5 % vs. 41.7 % vs. 20.2 %; prostate: 49.5 % vs. 38.0 % vs. 32.2 %), and higher use of cardiovascular medications. In breast cancer patients with LDL<100 mg/dl, chronic kidney disease was more common (21.6 % vs. 10.8 vs. 10.6 %), while hyperlipidemia (67.5 % vs. 74.4 % vs. 78.8 %) and valvular heart disease (9.4 % vs. 14.2 % vs. 19.7 %) were less common. In prostate cancer patients with LDL<100 mg/dl, higher rates of coronary artery disease (50.5 % vs. 42.9 % vs. 28.8 %), peripheral arterial disease (9.0 % vs. 3.3 % vs. 1.7 %), and atrial fibrillation (11.1 % vs. 3.3 vs. 6.8 %) were observed.

Laboratory data showed that patients with LDL<100 mg/dl had lower total cholesterol (breast: 166.8 vs. 191.2 vs. 217.4 mg/dl; prostate: 155.6 vs. 183.5 vs. 206.3 mg/dl) and lower eGFR. Breast cancer patients with LDL<100 mg/dl had higher HbA1C (6.4 % vs. 6.2 % vs. 6.0 %); prostate cancer patients with LDL<100 mg/dl had lower serum triglyceride (93.0 vs. 112.2 vs. 115.4 mg/dl). Cancer stages, locations, and treatment did not significantly differ by LDL levels. The median follow-up periods in both cancers were less than three years.

**Fig. 1:**
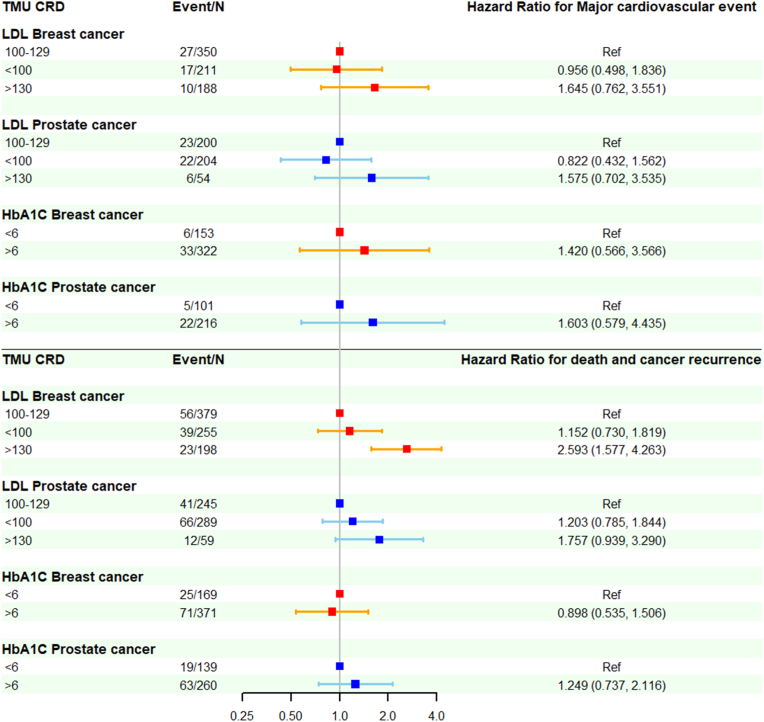
The association of major cardiovascular events, all-cause mortality, and cancer recurrence with serum low-density protein cholesterol and hemoglobulin A1C in patients with breast or prostate cancers: analysis from Taipei Medical University Clinical Research Database. Hazards ratios with 95 % confidence intervals were used to express the association of major cardiovascular events, all-cause mortality, and cancer recurrence with different levels of serum low-density protein cholesterol and hemoglobulin A1C. The multivariable Cox proportional hazard model with time-dependent covariates was used. Red lines indicate breast cancer. Blue lines indicate prostate cancer. HbA1C = hemoglobulin A1C; LDL = low-density protein cholesterol; TMU CRD = Taipei Medical University Clinical Research Database; Ref = reference. (For interpretation of the references to colour in this figure legend, the reader is referred to the Web version of this article.)

**Fig. 2:**
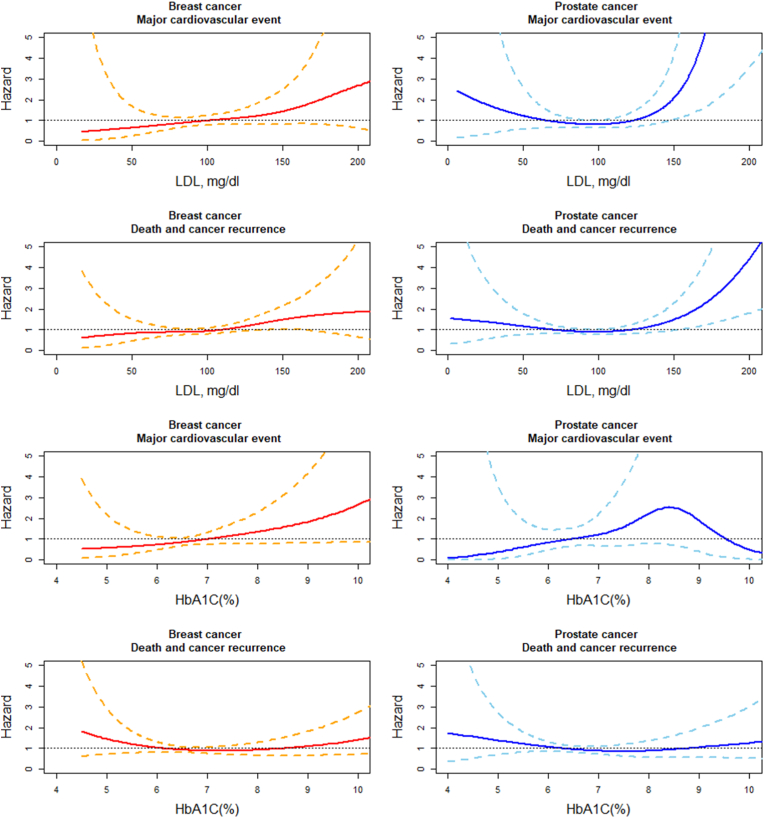
Multivariable adjusted hazard ratios for major cardiovascular events, all-cause mortality, and cancer recurrence according to levels of low-density lipoprotein cholesterol and hemoglobulin A1C on a continuous scale. Hazards ratios (solid lines) with 95 % confidence intervals (dashed lines) were calculated by the multivariable Cox proportional hazard model with time-dependent covariates was used. Red lines indicate breast cancer. Blue lines indicate prostate cancer. Black dash lines are the reference line, indicating no association with a hazard ratio of 1.0. HbA1C = hemoglobulin A1C; LDL = low-density protein cholesterol. (For interpretation of the references to colour in this figure legend, the reader is referred to the Web version of this article.)

In the crude Cox proportional hazard models, no significant association was observed between MACE and LDL levels. In the multivariable model with time-dependent covariates (Fig. 1; Supplemental Table 4), patients with LDL>130 mg/dl had a numerically but insignificant increased risk of MACE, compared to those with LDL 100–129 mg/dl (adjusted HR 1.65, 95 % CI: 0.76–3.55 in breast cancer; adjusted HR 1.58, 95 % CI: 0.70–3.54 in prostate cancer). For the composite outcomes of all-cause mortality and cancer recurrence (Fig. 1; Table 1), the crude models for breast cancer showed a U-shape association across the LDL levels, with the lowest risk in LDL 100–129 mg/dl (LDL <100 mg/dl: crude HR 1.57, 95 % CI: 1.01–2.45, p = 0.047; LDL>130 mg/dl: crude HR 1.95, 95 % CI: 1.20–3.18, p = 0.007). In the multivariable model, LDL>130 mg/dl remains a significantly increased risk, compared to LDL 100–129 mg/dl (adjusted HR 2.59, 95 % CI: 1.58–4.26, p < 0.001). In prostate cancer, the crude models showed no significant association across the LDL levels. In the multivariable models, LDL>130 mg/dl had a numerical but insignificant increased risk, compared to LDL 100–129 mg/dl (adjusted HR 1.76, 95 % CI 0.94–3.29, p = 0.08). The cubic spline plots suggested that, in breast cancer, higher LDL levels may be associated with higher risks of both MACE and the composite cancer outcome (all-cause mortality and cancer recurrence) (Fig. 2). In prostate cancer, a U-shape association was observed in both MACE and the composite cancer outcome.

**Table 1:** The association between low-density lipoprotein cholesterol and total death or cancer recurrence in breast and prostate cancer.

Breast Cancer							
TMU CRD							
LDL	Event/N	Crude Model	*p*-value	Adjusted Model 1^a^	*p*-value	Adjusted Model 2 b	*p*-value
100–129	59/379	Ref		Ref		Ref	
<100	39/255	1.57 [1.01, 2.45]	0.047	1.28 [0.82, 2.01]	0.27	1.15 [0.73, 1.82]	0.54
≥130	23/198	1.95 [1.20, 3.18]	0.007	2.28 [1.40, 3.73]	0.001	2.59 [1.58, 4.26]	<0.001

**TriNetX**^e^							
LDL	Event/N	Crude Model	*p*-value	PSM Model 1 c	*p*-value	PSM Model 2 d	*p*-value

100–129	11,219/92,586	Ref		Ref		Ref	
<100	16,466/107,076	1.34 [1.31, 1.37]	<0.001	1.24 [1.21, 1.27]	<0.001	1.13 [1.10, 1.16]	<0.001
≥130	8681/73,490	0.98 [0.95, 1.01]	0.13	1.01 [0.98, 1.04]	0.63	1.05 [1.02, 1.09]	<0.001

**Prostate Cancer**							
**TMU CRD**							
LDL	Event/N	Crude Model	*p*-value	Adjusted Model 1 a	*p*-value	Adjusted Model 2 e	P

100–129	41/245	Ref		Ref		Ref	
<100	66/289	1.40 [0.92, 2.13]	0.11	1.26 [0.83, 1.91]	0.29	1.20 [0.79, 1.84]	0.40
≥130	12/59	1.29 [0.70, 2.38]	0.42	1.72 [0.92, 3.19]	0.09	1.76 [0.94, 3.29]	0.08

**TriNetX**^g^							
LDL	Event/N	Crude Model	*p*-value	PSM Model 1 c	*p*-value	PSM Model 2 f	*p*-value

100–129	10,583/69,868	Ref		Ref		Ref	
<100	21,485/115,311	1.33 [1.30, 1.36]	<0.001	1.19 [1.16, 1.22]	<0.001	1.07 [1.04, 1.10]	<0.001
≥130	6296/42,545	0.98 [0.95, 1.01]	0.11	1.04 [1.00, 1.07]	0.048	1.08 [1.04, 1.12]	<0.001

The associations were expressed as hazard ratio with 95 % confidence intervals.

LDL = low-density lipoprotein cholesterol; PSM = Propensity score matching; TMU CRD = Taipei Medical University Clinical Research Database; Ref = reference.

a Adjusted Model 1 were estimated using time-dependent cox model adjusted for age.

b Adjusted Model 2 were estimated using time-dependent cox model adjusted for age, coronary artery disease, peripheral arterial disease, hypertension, diabetes mellitus, hyperlipidemia, atrial fibrillation, valvular heart disease, chronic lung disease, chronic kidney disease, surgery, year of cancer diagnosis, primary cancer site, anthracyclines, anti-HER2 Therapy, hormone therapy.

c PSM Model 1 were estimated using cox model and matched by propensity score for age.

d PSM Model 2 were estimated using cox model and matched by propensity score for age, coronary artery disease, peripheral arterial disease, hypertension, diabetes mellitus, hyperlipidemia, atrial fibrillation, valvular heart disease, chronic lung disease, chronic kidney disease, anti-HER2 therapy.

e Adjusted Model 2 were estimated using time-dependent cox model adjusted for age, coronary artery disease, peripheral arterial disease, hypertension, diabetes mellitus, hyperlipidemia, atrial fibrillation, valvular heart disease, chronic lung disease, chronic kidney disease, surgery, year of cancer diagnosis, primary cancer site, GnRH agonist, androgen synthesis inhibitor, androgen receptor blocker.

f PSM Model 2 were estimated using cox model and matched by propensity score for age, coronary artery disease, peripheral arterial disease, hypertension, diabetes mellitus, hyperlipidemia, atrial fibrillation, valvular heart disease, chronic lung disease, chronic kidney disease, GnRH agonist, androgen synthesis inhibitor, androgen receptor blocker.

g Information of cancer recurrence was unavailable. p-value for log-rank test.

We selected 540 females with breast cancer and 399 males with prostate cancer, each with ≥2 HbA1C measurements (Supplemental Tables 2 and 3). In the breast cancer, patients with HbA1C<6 % were younger and had lower rates of hypertension (56.8 % vs. 71.4 %), diabetes mellitus (20.7 % vs. 84.1 %), hyperlipidemia (59.2 % vs. 75.5 %) and use of cardiovascular medications. They also had lower body mass index (23.7 vs. 26.2 kg/m^2^), lower serum triglyceride (102.2 vs. 130.4 mg/dl), higher total cholesterol (194.2 vs.184.9 mg/dl), and higher serum high-density lipoprotein cholesterol (56.0 vs.49.0 mg/dl). In addition, they had lower rate of using aromatase inhibitor (33.7 % vs. 47.6 %). In the prostate cancer, patients with HbA1C<6 % had higher rate of atrial fibrillation (10.1 % vs. 3.8 %), but lower rate of diabetes mellitus (28.8 % vs. 81.9 %) and using anti-diabetic medications. They also had lower body mass index (24.2 vs. 25. 5 kg/m^2^), lower serum triglycerides (99.5 vs. 114.8 mg/dl), and higher serum high-density lipoprotein cholesterol (50.3 vs. 43.5 mg/dl). Cancer stages, locations, and treatment did not significantly differ between HbA1C levels in either cancer except aromatase inhibitor for breast cancer. In both crude and multivariable models, no significant association was observed between HbA1C levels and MACE or the composite cancer outcome (all-cause mortality and cancer recurrence) (Fig. 1; Supplemental Tables 5 and 6). In both cancers, the cubic spline plots suggested that higher HbA1C may be associated with increased MACE (Fig. 2). A U-shape association was observed in the composite cancer outcome.

**Fig. 3:**
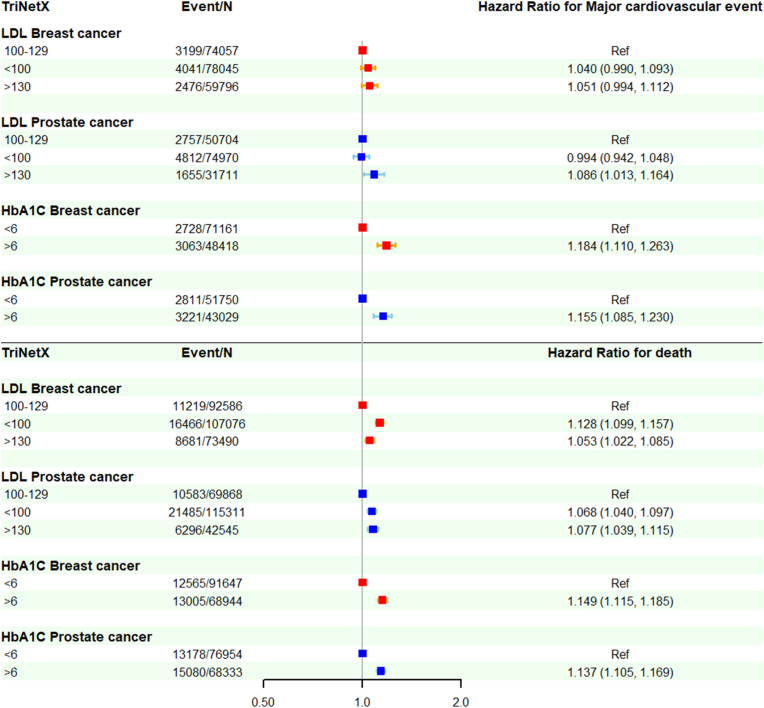
The association of major cardiovascular events, all-cause mortality, and cancer recurrence with serum low-density protein cholesterol and hemoglobulin A1C in patients with breast or prostate cancers: analysis from the TriNetX research network. Hazards ratios with 95 % confidence intervals were used to express the association of major cardiovascular events, all-cause mortality, and cancer recurrence with different levels of serum low-density protein cholesterol and hemoglobulin A1C. The Cox proportional hazard model with propensity score matching was used. Red lines indicate breast cancer. Blue lines indicate prostate cancer. HbA1C = hemoglobulin A1C; LDL = low-density protein cholesterol; Ref = reference. (For interpretation of the references to colour in this figure legend, the reader is referred to the Web version of this article.)

Given the small event numbers and short follow-up in the TMUCRD cohorts, we used the TriNetX platform to validate previous findings (Supplemental Figs. 3 and 4). In crude models for MACE, patients with LDL<100 mg/dl had a higher risk than patients with LDL 100–129 mg/dl (crude HR 1.26, 95 % CI: 1.20–1.32, p < 0.001 in breast cancer; crude HR 1.27, 95 % CI 1.21–1.33, p < 0.001 in prostate cancer). After PS-matching, the association became insignificant; but the risk of LDL>130 mg/dl became significantly higher compared to LDL 100–129 mg/dl in prostate cancer (PS-matching HR 1.09, 95 % CI: 1.01–1.16, p = 0.02) (Fig. 3; Supplemental Table 4). For all-cause mortality, a significant U-shape association was observed across LDL levels, with the lowest risk at LDL 100–129 mg/dl. This pattern appeared in both breast cancer (LDL<100 mg/dl: PS-matching HR 1.13, 95 % CI: 1.10–1.16, p < 0.001; LDL >130 mg/dl: PS-matching HR 1.05, 95 % CI: 1.02–1.09, p < 0.001) and prostate cancers (LDL<100 mg/dl: PS-matching HR 1.07, 95 % CI: 1.04–1.10, p < 0.001; LDL>130 mg/dl: PS-matching HR 1.08 95 % CI: 1.04–1.12, p < 0.001) (Fig. 3; Table 1). In contrast, both crude models and PS-matching models showed HbA1C>6 % was associated with higher risks of major cardiovascular risks (PS-matching HR 1.18, 95 % CI 1.110–1.26, p < 0.001 in breast cancer; PS-matching HR 1.16, 95 % CI 1.09–1.23, p < 0.001 in prostate cancer) and all-cause mortality (PS-matching HR 1.15, 95 % CI 1.12–1.19, p < 0.001 in breast cancer; PS-matching HR 1.14, 95 % CI 1.11–1.17, p < 0.001 in prostate cancer) (Fig. 3; Supplemental Tables 5 and 6).

## 4 Discussion

This study showed that in patients with breast and prostate cancer, a higher LDL level (≥130 mg/dL) may be associated with an increased risk of MACE, particularly in prostate cancer. A U-shaped association was observed between LDL levels and all-cause mortality in both breast and prostate cancers, with the lowest risk at the level of 100–129 mg/dL. An elevated HbA1C level (>6 %) was associated with a higher risk of MACE, with a potential U-shaped association for all-cause mortality. The strength of this study is the use of Cox proportional hazard models with time-dependent covariates to account for fluctuations in LDL and HbA1C, with the findings validated in a large multicenter electronic health record database.

This study revealed a U-shaped association between LDL levels and all-cause mortality in patients with breast and prostate cancer. This finding was consistent with previous population studies, especially in individuals not receiving cholesterol-lowering statins [7,8]. Elevated cholesterol, through intracellular cholesterol dysregulation, may contribute to cancer development or progression, including breast and prostate cancers [[25], [26], [27]]. Mouse models suggested that the cholesterol metabolite 27-hydroxycholesterol may promote cancer metastasis by inhibiting immune response [28]. However, whether reducing cholesterol improves cancer outcomes remain unclear. In contrast, very low LDL was reported to be associated with increased risks of all-cause mortality and cancer mortality, but not cardiovascular mortality [7,8]. However, this result should be interpreted cautiously. In patients with approved cardiovascular indications, randomized trials have shown that LDL-lowering statins reduce cardiovascular events without increasing cancer incidence or mortality [29,30]. Additionally, trials of proprotein convertase subtilisin/kexin type 9 inhibition showed that achieving very low LDL levels (<20 mg/dl) reduced cardiovascular events without long-term safety concerns [31]. Low LDL level may reflect severe chronic illness or frailty, rather than a direct cause of increased mortality. Recent studies suggest that cardiovascular risk was associated with cumulative duration of LDL exposure or statin use [32,33]. Because most observational studies assessed baseline LDL levels, further research, taking cumulative exposure into account, is needed to confirm the association between LDL exposure and all-cause mortality or cancer-related outcomes.

Elevated HbA1C has been associated with all-cause mortality in a U-shaped pattern in both diabetic and non-diabetic population [11,12]. Elevated HbA1C may also worsen cancer-specific outcomes, even in non-diabetic patients, with a potentially stronger effect observed in breast cancer than in prostate cancer [[16], [17], [18],^34^]. This study found that elevated HbA1C was associated with increased mortality in both breast and prostate cancers. Hyperglycemia may promote tumor growth and chemotherapy resistance [35]. Obesity, a risk factor for diabetes, may increase cancer incidence, progression, and mortality through insulin resistance, pro-tumorigenic circulating factors, and chronic inflammation [[36], [37], [38]]. One recent study suggested that glucagon-like peptide 1 receptor agonists, anti-diabetic drugs which help weight loss, may lower cancer incidence [39]. While intensive glucose management in patients with multiple comorbidities may increase the risk of hypoglycemia and all-cause mortality, further trials are needed to determine optimal glucose target and weight management for cancer survivors.

Previous studies have shown that elevated LDL [7] and HbA1C [13] were associated with increased cardiovascular risks in the general population. Recently, dyslipidemia [9,10] and diabetes [14,15] were considered an independent cardiovascular risk factor in both adult and child cancer survivors. Our findings were consistent with these observations. Although the TMUCRD models did not show significant association of CVD and all-cause mortality with LDL and HbA1C, the cubic spline plots suggested the trends toward higher risks. These trends were further validated in the larger TriNetX cohort, which showed significantly elevated risks. Notably, LDL<100 mg/dl was associated with an increased cardiovascular risk in the TMUCRD prostate cohort and the crude TriNetX models. However, the association disappeared after PS matching, suggesting a probable confounding effect because patients with high cardiovascular risks received more aggressive cholesterol management. In summary, our findings support blood sugar and lipid management based on current guidelines to reduce cardiovascular risks in cancer survivors [2,3].

### 4.1 Limitations

First, this retrospective study used single-center electronic medical records in Asia, validated by a large multicenter database, primarily from Western populations. Small sample size and short follow-up period may limit statistical power. Some confounders, such as medication adherence, dosing, lifestyles, and socioeconomic status, cannot be fully evaluated. However, the consistent trends observed in two diverse cohorts with different analytic designs offered a general understanding of the effects of LDL and HbA1C. This dual-cohort design enhances generalizability and highlights the value of regional data in guiding targeted interventions. Second, LDL or HbA1C in this study were not measured longitudinally. The laboratory assays and methodologies may vary over time across institutions. Besides, many patients were excluded during the selection process. However, the TMUCRD cohort included patients with at least two measurements and adjusted for time-dependent covariate to minimize time-related variability. Although the built-in functions of the TriNetX network limited longitudinal assessment and may lead to repeated patient inclusion, propensity score matching helped address confounding by indication of testing LDL or HbA1C. This study mitigated the limitation of only using baseline measurements. Third, detailed information of cancer stages, recurrence, and cause-specific mortality was not fully available in the TMHCRD and TriNetX databases. Our findings may not entirely represent cancer-specific outcomes. Instead of using difficult-to-validate cancer outcomes, this study focused on all-cause mortality to reduce the impact of competing risks from potential comorbidities. Additionally, this study adjusted cancer treatment, which reflected underlying cancer severity, addressing treatment-associated risks in the absence of cancer staging. Fourth, requiring at least two LDL or HbA1C measurements may introduce healthy survivor bias, potentially underestimating risks in sicker patients who were less likely to receive repeated testing. Fifth, competing risks from cancer-related death could not be evaluated when cause-specific mortalities data were not available. The above limitations highlight the need for prospective studies with standardized laboratory protocols, longitudinal measurements, and longer follow-up periods to assess the impact of LDL and HbA1C on cancer-related outcomes. Sixth, cancer types provide different cardiovascular risks or prognosis. To reduced cancer-related heterogeneity, this study focused on cancers with a longer survival, specifically breast cancer and prostate cancers, to observe the effects of LDL and HbA1C. Since there were famous concepts of “CVD-Diabetes Mellitus-Cancer” strips proposed by Chinese scientists [40], all patients with breast and prostate cancer should have a comprehensive iRT-ABCDEFG program [41,42] and healthier lifestyle [43] for better control of risk factors and prevention of adverse events and outcomes, particularly the “CVD-Diabetes Mellitus-Cancer” strips.

## 5 Conclusions

This study suggested that in patients with breast and prostate cancer, higher LDL levels may be associated with an increased risk of MACE, with a U-shaped association with all-cause mortality. Elevated HbA1C was associated with increased risks of MACE and all-cause mortality.

## CRediT authorship contribution statement

**Yen-Chou Chen:** Writing – original draft, Visualization, Methodology, Funding acquisition, Conceptualization. **Jhih-Yuan Lu:** Visualization, Methodology, Formal analysis. **Chun-Yao Huang:** Writing – review & editing, Supervision, Methodology, Conceptualization. **Yu-Hsuan Joni Shao:** Writing – review & editing, Supervision, Methodology, Formal analysis.

## Data availability

Data sharing is not applicable. This is a retrospective study using deidentified and delinked electronic medical record databases. No new data were generated or analyzed in support of this research.

## Declaration of competing interest

The authors report no relationships that could be construed as a conflict of interest.
