# Supplementary material for: Associations of low-density lipoprotein cholesterol and hemoglobin A1C with cardiovascular events and mortality in breast and prostate cancer patients
Source: Int J Cardiol Cardiovasc Risk Prev. 2025 Jul 11;26:200468. doi: 10.1016/j.ijcrp.2025.200468 (PMC12281014; doi:10.1016/j.ijcrp.2025.200468)
Supplement: Multimedia component 1 [file mmc1.docx]

**Supplemental Table 1. Codes of International Classification of Diseases Used for Cardiovascular Events**

| **Incident** | **ICD-10 CM** | **ICD-9 CM** |
| --- | --- | --- |
| **Heart Failure** | I11.0, I13.0, I13.2, I42, I50 | 402.01, 402.11, 402.91, 404.01, 404.03, 404.11, 404.13, 404.91, 404.93, 425, 428, 785.51 |
| **Coronary Artery Disease** | I20, I23-I25 | 411-414 |
|  |  |  |
| **Myocardial Infarction** | I21, I22 | 410 |
| **Sudden cardiac death** | I46.9, R99 | 427.5, 798, 798.1, 798.2 |
| **Cerebral Ischemic Stroke** | I63, I65, I66 | 433, 434, 436 |
| **Cerebral Hemorrhagic Stroke** | I60, I61, I62 | 430, 431, 432 |
| **Peripheral Artery Disease** | I70.2, I70.9, I73.9, I75.0 | 440.2, 443.9 |
| **Valvular Heart Disease** | I34-I39 | 424 |
| **Atrial Fibrillation** | I48 | 427.3 |
| **Hypertension** | I10-I15 | 401-405 |
| **Diabetes Mellitus** | E08-E13 | 250 |
| **Dyslipidemia** | E78 | 272 |
| **Chronic Obstructive Pulmonary Disease** | J41-J47, J60-J66 | 491-496, 500-505 |
| **Chronic Kidney Disease** | E10.2, E11.2, I12, I13, M10.3, N00-N08, N14, N17-19, N25-27, Q60, Q61 | 250.4, 274.1, 580-589, 403.x1, 404.x2, 404.x3 |

ICD-10 CM = International Classification of Diseases, Tenth Revision, Clinical Modification; ICD-9 CM = International Classification of Diseases, Ninth Revision, Clinical Modification.

**Supplemental Table 2. Characteristics of participants with breast cancer, stratified by low-density lipoprotein cholesterol and hemoglobin A1C, respectively.**

|  | **LDL<100** | **LDL100-129** | **LDL≥130** |  | **HbA1C <6** | **HbA1C ≥6** |  |
| --- | --- | --- | --- | --- | --- | --- | --- |
|  | **(n =255)** | **(n = 379)** | **(n = 198)** | **p-value** | **(n =169)** | **(n = 371)** | **p-value** |
| **Age (year)** | 67.2 [58.4, 77.2] | 63.8 [57.5, 73.6] | 59.8 [53.2, 66.8] | <0.001 | 61.5 [52.5, 70.7] | 66.2 [59.2, 75.7] | <0.001 |
| 20-39 | 6 (2.4) | 5 (1.3) | 3 (1.5) | <0.001 | 6 (3.0) | 0 (0.0) | <0.001 |
| 40-54 | 41 (16.1) | 67 (17.7) | 63 (31.8) |  | 53 (31.4) | 49 (13.2) |  |
| 55-64 | 60 (23.5) | 132 (34.8) | 69 (34.8) |  | 42 (24.9) | 124 (33.4) |  |
| 65-74 | 66 (25.9) | 94 (24.8) | 47 (23.7) |  | 37 (21.9) | 97 (26.1) |  |
| 75-84 | 59 (23.1) | 70 (18.5) | 14 (7.1) |  | 20 (11.8) | 84 (22.6) |  |
| ≥85 | 23 (9.0) | 11 (2.9) | 2 (1.0) |  | 12 (7.1) | 17 (4.6) |  |
| **Body Mass Index (kg/m^2^)** | 24.6 [22.3, 28.3] | 25.2 [22.2, 28.0] | 24.8 [22.7, 27.5] | 0.944 | 23.7 [21.6, 26.9] | 26.2 [23.4, 29.0] | <0.001 |
| <30 (kg/m^2^) | 196 (83.9) | 294 (87.0) | 155 (89.1) | 0.304 | 139 (89.7) | 266 (79.9) | 0.011 |
| ≥30 | 37 (16.1) | 44 (13.0) | 19 (10.9) |  | 16 (10.3) | 67 (20.1) |  |
| **Clinical Stage** |  |  |  | 0.526 |  |  | 0.076 |
| 0 | 22 (11.6) | 30 (11.4) | 18 (12.2) |  | 15 (12.8) | 28 (10.4) |  |
| 1 | 61 (32.3) | 95 (36.0) | 63 (42.9) |  | 45 (38.5) | 81 (30.2) |  |
| 2 | 86 (45.5) | 111 (42.0) | 52 (35.4) |  | 51 (43.6) | 120 (44.8) |  |
| 3 | 9 (4.8) | 11 (4.2) | 9 (6.1) |  | 3 (2.6) | 15 (5.6) |  |
| 4 | 11 (5.8) | 17 (6.4) | 5 (3.4) |  | 3 (2.6) | 24 (9.0) |  |
| **Pathological Stage** |  |  |  | 0.213 |  |  | 0.613 |
| 0 | 11 (7.4) | 10 (4.8) | 9 (7.8) |  | 6 (6.5) | 10 (4.8) |  |
| 1 | 52 (35.1) | 88 (42.1) | 58 (50.0) |  | 38 (41.3) | 77 (36.8) |  |
| 2 | 64 (43.2) | 83 (39.7) | 32 (27.6) |  | 38 (41.3) | 85 (40.7) |  |
| 3 | 16 (10.8) | 21 (10.0) | 15 (12.9) |  | 8 (8.7) | 28 (13.4) |  |
| 4 | 5 (3.4) | 7 (3.3) | 2 (1.7) |  | 2 (2.2) | 9 (4.3) |  |
| **Primary Cancer Site** |  |  |  | 0.951 |  |  | 0.730 |
| Left | 85 (33.3) | 130 (34.3) | 71 (35.9) |  | 60 (35.3) | 119 (31.5) |  |
| Right | 86 (33.7) | 126 (33.2) | 68 (34.3) |  | 56 (32.9) | 123 (32.5) |  |
| Bilateral | 2 (0.8) | 6 (1.6) | 3 (1.5) |  | 52 (30.6) | 129 (34.1) |  |
| Unknown | 82 (32.2) | 117 (30.9) | 56 (28.3) |  | 2 (1.2) | 7 (1.9) |  |
| **Year of Cancer Diagnosis** |  |  |  | 0.220 |  |  | 0.001 |
| 2011 | 1 (0.4) | 8 (2.1) | 2 (1.0) |  | 1 (0.6) | 6 (1.6) |  |
| 2012 | 11 (4.3) | 16 (4.2) | 3 (1.5) |  | 1 (0.6) | 16 (4.3) |  |
| 2013 | 13 (5.1) | 21 (5.5) | 12 (6.1) |  | 4 (2.4) | 18 (4.9) |  |
| 2014 | 20 (7.8) | 34 (9.0) | 16 (8.1) |  | 6 (3.6) | 36 (9.7) |  |
| 2015 | 22 (8.6) | 43 (11.3) | 26 (13.1) |  | 14 (8.3) | 44 (11.9) |  |
| 2016 | 32 (12.5) | 41 (10.8) | 33 (16.7) |  | 25 (14.8) | 46 (12.4) |  |
| 2017 | 40 (15.7) | 55 (14.5) | 24 (12.1) |  | 20 (11.8) | 55 (14.8) |  |
| 2018 | 40 (15.7) | 80 (21.1) | 31 (15.7) |  | 32 (18.9) | 63 (17.0) |  |
| 2019 | 50 (19.6) | 58 (15.3) | 35 (17.7) |  | 44 (26.0) | 68 (18.3) |  |
| 2020 | 26 (10.2) | 23 (6.1) | 16 (8.1) |  | 22 (13.0) | 19 (5.1) |  |
| **History of Major Cardiovascular Event** |  |  |  |  |  |  |  |
| Heart Failure | 36 (14.1) | 32 (8.4) | 12 (6.1) | 0.009 | 15 (8.9) | 43 (11.6) | 0.427 |
| Myocardial Infarction^a^ | 3 (1.2) | 4 (1.1) | 1 (0.5) | 0.816 | 1 (0.6) | 5 (1.3) | 0.671 |
| Cerebral Ischemic Stroke | 32 (12.5) | 31 (8.2) | 8 (4.0) | 0.005 | 17 (10.1) | 39 (10.5) | 0.994 |
| **Cardiovascular Comorbidity** |  |  |  |  |  |  |  |
| Coronary Artery Disease | 94 (36.9) | 118 (31.1) | 56 (28.3) | 0.127 | 52 (30.8) | 114 (30.7) | 1.000 |
| Peripheral Arterial Disease | 12 (4.7) | 8 (2.1) | 3 (1.5) | 0.070 | 5 (3.0) | 9 (2.4) | 0.945 |
| Hypertension | 183 (71.8) | 241 (63.6) | 88 (44.4) | <0.001 | 96 (56.8) | 265 (71.4) | 0.001 |
| Diabetes Mellitus | 134 (52.5) | 158 (41.7) | 40 (20.2) | <0.001 | 35 (20.7) | 312 (84.1) | <0.001 |
| Hyperlipidemia | 172 (67.5) | 282 (74.4) | 156 (78.8) | 0.021 | 100 (59.2) | 280 (75.5) | <0.001 |
| Atrial Fibrillation | 9 (3.5) | 6 (1.6) | 1 (0.5) | 0.054 | 4 (2.4) | 7 (1.9) | 0.970^a^ |
| Valvular Heart Disease | 24 (9.4) | 54 (14.2) | 39 (19.7) | 0.008 | 24 (14.2) | 34 (9.2) | 0.109 |
| Chronic Lung Disease | 46 (18.0) | 49 (12.9) | 23 (11.6) | 0.096 | 26 (15.4) | 59 (15.9) | 0.979 |
| Chronic Kidney Disease | 55 (21.6) | 41 (10.8) | 21 (10.6) | <0.001 | 23 (13.6) | 76 (20.5) | 0.073 |
| **Laboratory Data** |  |  |  |  |  |  |  |
| HbA1C (%) | 6.4 [5.9, 7.1] | 6.2 [5.8, 7.0] | 6.0 [5.6, 6.5] | 0.001 | - | - | - |
| HbA1C <6 | 66 (32.8) | 95 (36.7) | 56 (51.9) | 0.004 | - | - | - |
| HbA1C ≥6 | 135 (67.2) | 164 (63.3) | 52 (48.1) |  | - | - | - |
| LDL (mg/dl) |  |  |  | - | 108.5 [95.1, 130.7] | 104.7 [91.6, 119.6] | 0.067 |
| LDL <100 | - | - | - | - | 51 (31.9) | 145 (40.3) | 0.011 |
| LDL 100-129 | - | - | - |  | 68 (42.5) | 161 (44.7) |  |
| LDL ≥130 | - | - | - |  | 41 (25.6) | 54 (15.0) |  |
| Total Cholesterol(mg/dl) | 166.8 [154.7, 179.8] | 191.2 [179.0, 204.1] | 217.4 [207.2, 235.5] | <0.001 | 194.2 [174.0, 208.3] | 184.9 [165.9, 203.4] | 0.013 |
| Serum Triglyceride(mg/dl) | 108.00 [81.3, 152.5] | 110.8 [85.7, 151.0] | 119.5 [86.1, 155.5] | 0.271 | 102.2 [79.1, 138.7] | 130.4 [98.5, 180.2] | <0.001 |
| Serum creatinine(mg/dl) | 0.72 [0.62, 0.90] | 0.68 [0.60, 0.80] | 0.68 [0.60, 0.76] | 0.002 | 0.70 [0.62, 0.81] | 0.69 [0.60, 0.84] | 0.765 |
| eGFR (CKD-EPI, mL/min per 1.73 m²) | 85.6 [60.7, 103.8] | 94.9 [77.7, 111.4] | 95.6 [82.7, 110.4] | <0.001 | 94.3 [78.1, 106.8] | 88.8 [67.4, 110.0] | 0.513 |
| High-density Lipoprotein Cholesterol(mg/dl) | 52.7 [45.6, 63.9] | 53.0 [45.6, 62.5] | 56.0 [46.4, 66.5] | 0.354 | 56.0 [48.5, 66.7] | 49.0 [42.2, 57.7] | <0.001 |
| **Cardiovascular Medication** |  |  |  |  |  |  |  |
| Renin-angiotensin-aldosterone system Inhibitor | 138 (54.1) | 147 (38.8) | 48 (24.2) | <0.001 | 64 (37.9) | 189 (50.9) | 0.006 |
| Calcium Channel Blocker | 94 (36.9) | 123 (32.5) | 37 (18.7) | <0.001 | 48 (28.4) | 135 (36.4) | 0.085 |
| β Blocker | 92 (36.1) | 130 (34.3) | 48 (24.2) | 0.017 | 39 (23.1) | 135 (36.4) | 0.003 |
| Mineralocorticoid receptor antagonists | 13 (5.1) | 14 (3.7) | 1 (0.5) | 0.024 | 7 (4.1) | 10 (2.7) | 0.531 |
| Loop Diuretics | 31 (12.2) | 25 (6.6) | 7 (3.5) | 0.002 | 10 (5.9) | 51 (13.7) | 0.012 |
| Thiazides Diuretics | 20 (7.8) | 16 (4.2) | 1 (0.5) | 0.001 | 7 (4.1) | 21 (5.7) | 0.597 |
| Anti-platelet | 24 (9.3) | 23 (6.0) | 6 (3.0) | 0.022 | 13 (7.7) | 27 (7.3) | 1.000 |
| Oral Anti-coagulant | 7 (2.7) | 11 (2.9) | 1 (0.5) | 0.157 | 6 (3.6) | 6 (1.6) | 0.272 |
| Statin | 139 (54.5) | 178 (47.0) | 78 (39.4) | 0.006 | 58 (34.3) | 211 (56.9) | <0.001 |
| Insulin | 49 (19.2) | 41 (10.8) | 8 (4.0) | <0.001 | 6 (3.6) | 96 (25.9) | <0.001 |
| Metformin | 89 (34.9) | 93 (24.5) | 19 (9.6) | <0.001 | 8 (4.7) | 205 (55.3) | <0.001 |
| Sulfonylurea | 46 (18.0) | 45 (11.9) | 8 (4.0) | <0.001 | 2 (1.2) | 104 (28.0) | <0.001 |
| Thiazolidine^a^ | 8 (3.1) | 2 (0.5) | 1 (0.5) | 0.016 | 0 (0.0) | 12 (3.2) | 0.022 |
| Sodium-glucose cotransporter-2 Inhibitor^a^ | 3 (1.2) | 7 (1.8) | 3 (1.5) | 0.935 | 1 (0.6) | 11 (3.0) | 0.116 |
| Dipeptidyl-peptidase 4 Inhibitor | 56 (22.0) | 41 (10.8) | 8 (4.0) | <0.001 | 2 (1.2) | 113 (30.5) | <0.001^a^ |
| Glucagon-like peptide-1 receptor agonist^a^ | 5 (2.0) | 2 (0.5) | 1 (0.5) | 0.170 | 0 (0.0) | 7 (1.9) | 0.105 |
| Amiodarone | 10 (3.9) | 5 (1.3) | 1 (0.5) | 0.016 | 4 (2.4) | 10 (2.7) | 1.000^a^ |
| Meglitinide | 14 (5.5) | 7 (1.8) | 0 (0.0) | 0.001 | 1 (0.6) | 23 (6.2) | 0.007^a^ |
| **Cancer Therapy** |  |  |  |  |  |  |  |
| Surgery |  |  |  | 0.079 |  |  | 0.397 |
| None | 134 (52.5) | 200 (52.8) | 93 (47.0) |  | 90 (52.9) | 197 (52.1) |  |
| Breast-conserving Surgery | 44 (17.3) | 92 (24.3) | 48 (24.2) |  | 39 (22.9) | 72 (19.0) |  |
| Radical Mastectomy | 77 (30.2) | 87 (23.0) | 57 (28.8) |  | 41 (24.1) | 109 (28.8) |  |
| Radiation | 70 (27.5) | 114 (30.0) | 69 (34.8) | 0.231 | 50 (29.6) | 111 (29.8) | 1.000 |
| Anthracyclines^b^ | 60 (23.5) | 97 (25.6) | 58 (29.3) | 0.376 | 41 (24.1) | 96 (25.4) | 0.831 |
| Anti-HER2 Therapy^c^ | 55 (21.6) | 79 (20.8) | 51 (25.8) | 0.385 | 40 (23.5) | 82 (21.7) | 0.714 |
| Aromatase inhibitor^d^ | 116 (45.5) | 150 (39.5) | 70 (35.4) | 0.083 | 57 (33.7) | 177 (47.6) | 0.003 |
| Selective estrogen receptor modulator^e^ | 42 (16.5) | 55 (14.5) | 38 (19.2) | 0.341 | 28 (16.6) | 63 (16.9) | 1.000 |

Median (interquartile range) or n (%). P-values were calculated by Wilcoxon rank-sum test, Kruskal-Wallis’s test, or Chi-square test unless marked by superscript letter. Patients were stratified by the average values of serum low-density lipoprotein cholesterol and hemoglobin A1C before the cancer diagnosis.

a. P-values were calculated by Fisher’s exact test.

b. Anthracycline indicates doxorubicin and epirubicin. The equivalent dose of anthracycline is calculated by multiplying total doses by 1 for doxorubicin and 0.5 for epirubicin.

c. Anti-HER2 therapy indicates trastuzumab, pertuzumab, lapatinib.

d. Aromatase inhibitor indicates anastrozole, letrozole, exemestane.

e. Selective estrogen receptor modulator indicates tamoxifen, toremifene.

CKD-EPI = Chronic Kidney Disease Epidemiology Collaboration; eGFR = estimated glomerular filtration rate; HbA1C = hemoglobin A1C; HER2 = human epidermal growth factor receptor 2; LDL = low-density lipoprotein cholesterol.

**Supplemental Table 3. Characteristics of participants with prostate cancer, stratified by low-density lipoprotein cholesterol and hemoglobin A1C, respectively.**

|  | **LDL<100** | **LDL100-129** | **LDL≥130** |  | **HbA1C <6** | **HbA1C ≥6** |  |
| --- | --- | --- | --- | --- | --- | --- | --- |
|  | **(n =289)** | **(n = 245)** | **(n = 59)** | **p-value** | **(n = 139)** | **(n = 260)** | **p-value** |
| **Age (year)** | 76.8 [70.0, 84.0] | 72.5 [66.3, 80.8] | 68.4 [64.7, 75.1] | <0.001 | 75.2 [66.9, 82.4] | 73.5 [67.8, 81.3] | 0.717 |
| 20-39 | 0 (0.0) | 0 (0.0) | 0 (0.0) | <0.001 | 0 (0.0) | 0 (0.0) | 0.092 |
| 40-54 | 1 (0.3) | 5 (2.0) | 1 (1.7) |  | 4 (2.9) | 2 (0.8) |  |
| 55-64 | 33 (11.4) | 44 (18.0) | 15 (25.4) |  | 25 (18.0) | 37 (14.2) |  |
| 65-74 | 90 (31.1) | 90 (36.7) | 28 (47.5) |  | 40 (28.8) | 102 (39.2) |  |
| 75-84 | 102 (35.3) | 69 (28.2) | 13 (22.0) |  | 42 (30.2) | 81 (31.2) |  |
| ≥85 | 63 (21.8) | 37 (15.1) | 2 (3.4) |  | 28 (20.1) | 38 (14.6) |  |
| **Body Mass Index (kg/m^2^)** | 24.6 [22.5, 26.9] | 24.8 [23.0, 27.6] | 24.5 [22.5, 27.1] | 0.310 | 24.2 [22.1, 26.6] | 25.5 [23.4, 27.8] | 0.003 |
| BMI <30 (kg/m^2^) | 263 (93.6) | 219 (92.4) | 54 (96.4) | 0.541 | 125 (92.6) | 226 (91.9) | 0.959 |
| BMI ≥30 | 18 (6.4) | 18 (7.6) | 2 (3.6) |  | 10 (7.4) | 20 (8.1) |  |
| **Clinical Stage** |  |  |  | 0.970 |  |  | 0.312 |
| 0 | 2 (1.0) | 3 (1.7) | 1 (2.2) |  | 0 (0.0) | 5 (2.6) |  |
| 1 | 34 (16.3) | 29 (16.1) | 8 (17.4) |  | 17 (16.7) | 28 (14.7) |  |
| 2 | 89 (42.8) | 75 (41.7) | 17 (37.0) |  | 45 (44.1) | 76 (40.0) |  |
| 3 | 40 (19.2) | 39 (21.7) | 8 (17.4) |  | 16 (15.7) | 42 (22.1) |  |
| 4 | 43 (20.7) | 34 (18.9) | 12 (26.1) |  | 24 (23.5) | 39 (20.5) |  |
| **Pathological Stage** |  |  |  | 0.831 |  |  | 0.721 |
| 0 | 0 (0.0) | 1 (1.9) | 0 (0.0) |  | 0 (0.0) | 0 (0.0) |  |
| 1 | 3 (5.9) | 6 (11.1) | 1 (8.3) |  | 1 (3.6) | 5 (10.9) |  |
| 2 | 27 (52.9) | 21 (38.9) | 7 (58.3) |  | 15 (53.6) | 22 (47.8) |  |
| 3 | 15 (29.4) | 20 (37.0) | 3 (25.0) |  | 9 (32.1) | 15 (32.6) |  |
| 4 | 6 (11.8) | 6 (11.1) | 1 (8.3) |  | 3 (10.7) | 4 (8.7) |  |
| **Primary Cancer Site^a^** |  |  |  | 0.759 |  |  | 1.000 |
| Left | 1 (0.3) | 2 (0.8) | 0 (0.0) |  | 1 (0.7) | 3 (1.2) |  |
| Right | 1 (0.3) | 2 (0.8) | 0 (0.0) |  | 1 (0.7) | 2 (0.8) |  |
| Unknown | 287 (99.3) | 240 (98.0) | 59 (100.0) |  | 137 (98.6) | 255 (98.1) |  |
| **Year of Cancer Diagnosis** |  |  |  | 0.047 |  |  | 0.003^a^ |
| 2011 | 2 (0.7) | 1 (0.4) | 1 (1.7) |  | 0 (0.0) | 3 (1.2) |  |
| 2012 | 9 (3.1) | 13 (5.3) | 3 (5.1) |  | 1 (0.7) | 8 (3.1) |  |
| 2013 | 16 (5.5) | 17 (6.9) | 11 (18.6) |  | 2 (1.4) | 17 (6.5) |  |
| 2014 | 22 (7.6) | 22 (9.0) | 4 (6.8) |  | 10 (7.2) | 31 (11.9) |  |
| 2015 | 22 (7.6) | 29 (11.8) | 6 (10.2) |  | 6 (4.3) | 25 (9.6) |  |
| 2016 | 43 (14.9) | 19 (7.8) | 3 (5.1) |  | 12 (8.6) | 32 (12.3) |  |
| 2017 | 37 (12.8) | 34 (13.9) | 8 (13.6) |  | 24 (17.3) | 37 (14.2) |  |
| 2018 | 55 (19.0) | 42 (17.1) | 6 (10.2) |  | 35 (25.2) | 34 (13.1) |  |
| 2019 | 58 (20.1) | 53 (21.6) | 12 (20.3) |  | 39 (28.1) | 55 (21.2) |  |
| 2020 | 25 (8.7) | 15 (6.1) | 5 (8.5) |  | 10 (7.2) | 18 (6.9) |  |
| **History of Major Cardiovascular Event** |  |  |  |  |  |  |  |
| Heart Failure | 55 (19.0) | 33 (13.5) | 3 (5.1) | 0.014 | 20 (14.4) | 29 (11.2) | 0.437 |
| Myocardial Infarction | 8 (2.7) | 5 (2.0) | 0 (0.0) | 0.407 | 4 (2.9) | 8 (3.1) | 1.000^a^ |
| Cerebral Ischemic Stroke | 72 (24.9) | 43 (17.6) | 4 (6.8) | 0.003 | 35 (25.2) | 46 (17.7) | 0.101 |
| **Cardiovascular Comorbidity** |  |  |  |  |  |  |  |
| Coronary Artery Disease | 146 (50.5) | 105 (42.9) | 17 (28.8) | 0.006 | 58 (41.7) | 100 (38.5) | 0.598 |
| Peripheral Arterial Disease | 26 (9.0) | 8 (3.3) | 1 (1.7) | 0.007 | 10 (7.2) | 18 (6.9) | 1.000 |
| Hypertension | 232 (80.3) | 184 (75.1) | 29 (49.2) | <0.001 | 100 (71.9) | 205 (78.8) | 0.154 |
| Diabetes Mellitus | 143 (49.5) | 93 (38.0) | 19 (32.2) | 0.006 | 40 (28.8) | 213 (81.9) | <0.001 |
| Hyperlipidemia | 189 (65.4) | 164 (66.9) | 39 (66.1) | 0.932 | 93 (66.9) | 179 (68.8) | 0.777 |
| Atrial Fibrillation | 32 (11.1) | 8 (3.3) | 4 (6.8) | 0.003 | 14 (10.1) | 10 (3.8) | 0.023 |
| Valvular Heart Disease | 36 (12.5) | 24 (9.8) | 4 (6.8) | 0.355 | 17 (12.2) | 20 (7.7) | 0.191 |
| Chronic Lung Disease | 78 (27.0) | 65 (26.5) | 21 (35.6) | 0.354 | 35 (25.2) | 52 (20.0) | 0.286 |
| Chronic Kidney Disease | 92 (31.8) | 61 (24.9) | 13 (22.0) | 0.115 | 36 (25.9) | 82 (31.5) | 0.289 |
| **Laboratory Data** |  |  |  |  |  |  |  |
| HbA1C (%) | 6.1 [5.8, 6.7] | 6.1 [5.7, 6.7] | 6.3 [5.8, 7.4] | 0.435 | - | - | - |
| HbA1C <6 | 96 (42.3) | 74 (43.5) | 14 (41.2) | 0.953 | - | - | - |
| HbA1C ≥6 | 131 (57.7) | 96 (56.5) | 20 (58.8) |  | - | - |  |
| LDL (mg/dl) | - | - | - | - | 94.6 [83.1, 107.9] | 98.0 [85.8, 112.7] | 0.166 |
| LDL <100 | - | - | - | - | 80 (58.0) | 128 (54.0) | 0.518 |
| LDL 100-129 | - | - | - |  | 47 (34.1) | 94 (39.7) |  |
| LDL ≥130 | - | - | - |  | 11 (8.0) | 15 (6.3) |  |
| Total Cholesterol (mg/dl) | 155.6 [140.9, 169.2] | 183.5 [169.8, 194.6] | 206.3 [191.0, 222.6] | <0.001 | 165.9 [152.7, 184.1] | 167.9 [149.7, 187.2] | 0.792 |
| Serum Triglyceride (mg/dl) | 93.0 [71.6, 136.4] | 112.2 [83.8, 145.1] | 115.4 [90.5, 162.5] | <0.001 | 99.5 [72.9, 130.8] | 114.8 [84.0, 161.7] | <0.001 |
| Serum creatinine (mg/dl) | 1.05 [0.92, 1.28] | 1.00 [0.87, 1.17] | 1.03 [0.85, 1.13] | 0.009 | 1.03 [0.90, 1.18] | 1.05 [0.88, 1.29] | 0.269 |
| eGFR (mL/min per 1.73 m²) | 72.3 [54.1, 87.0] | 77.4 [59.1, 92.4] | 76.7 [69.4, 94.9] | 0.006 | 76.6 [63.8, 88.9] | 71.5 [54.2, 90.8] | 0.117 |
| High-density Lipoprotein Cholesterol (mg/dl) | 45.9 [39.0, 58.0] | 46.9 [41.5, 53.0] | 46.7 [42.0, 53.9] | 0.886 | 50.3 [41.8, 62.0] | 43.5 [37.1, 51.1] | <0.001 |
| PSA (ng/ml) | 8.0 [2.7, 20.7] | 7.1 [2.6, 13.3] | 9.7 [0.8, 22.8] | 0.327 | 6.6 [2.3, 12.9] | 7.7 [3.3, 22.8] | 0.217 |
| **Cardiovascular Medication** |  |  |  |  |  |  |  |
| Renin-angiotensin-aldosterone system Inhibitor | 183 (63.3) | 127 (51.8) | 24 (40.7) | 0.001 | 75 (54.0) | 144 (55.4) | 0.867 |
| Calcium Channel Blocker | 142 (49.1) | 112 (45.7) | 18 (30.5) | 0.033 | 52 (37.4) | 115 (44.2) | 0.227 |
| β Blocker | 117 (40.5) | 86 (35.1) | 13 (22.0) | 0.023 | 49 (35.3) | 91 (35.0) | 1.000 |
| Mineralocorticoid receptor antagonists | 14 (4.8) | 13 (5.3) | 0 (0.0) | 0.203 | 5 (3.6) | 11 (4.2) | 0.968 |
| Loop Diuretics | 93 (32.2) | 52 (21.2) | 6 (10.2) | <0.001 | 36 (25.9) | 62 (23.8) | 0.740 |
| Thiazides Diuretics | 19 (6.6) | 16 (6.5) | 4 (6.8) | 0.998 | 7 (5.0) | 20 (7.7) | 0.425 |
| Anti-platelet | 70 (24.2) | 44 (18.0) | 5 (8.5) | 0.013 | 40 (28.8) | 40 (15.4) | 0.002 |
| Oral Anti-coagulant | 25 (8.7) | 10 (4.1) | 3 (5.1) | 0.090 | 10 (7.2) | 18 (6.9) | 1.000 |
| Statin | 171 (59.2) | 111 (45.3) | 24 (40.7) | 0.001 | 65 (46.8) | 138 (53.1) | 0.273 |
| Insulin | 80 (27.7) | 36 (14.7) | 7 (11.9) | <0.001 | 15 (10.8) | 107 (41.2) | <0.001 |
| Metformin | 87 (30.1) | 49 (20.0) | 10 (16.9) | 0.009 | 13 (9.4) | 138 (53.1) | <0.001 |
| Sulfonylurea | 42 (14.5) | 27 (11.0) | 6 (10.2) | 0.398 | 4 (2.9) | 76 (29.2) | <0.001 |
| Thiazolidine^a^ | 4 (1.4) | 2 (0.8) | 1 (1.7) | 0.627 | 0 (0.0) | 6 (2.3) | 0.096 |
| Sodium-glucose cotransporter-2 Inhibitor^a^ | 5 (1.7) | 1 (0.4) | 1 (1.7) | 0.295 | 0 (0.0) | 7 (2.7) | 0.101 |
| Dipeptidyl-peptidase 4 Inhibitor | 49 (17.0) | 24 (9.8) | 4 (6.8) | 0.016 | 9 (6.5) | 71 (27.3) | <0.001 |
| Glucagon-like peptide-1 receptor agonist^a^ | 1 (0.3) | 0 (0.0) | 0 (0.0) | 1.000 | 0 (0.0) | 1 (0.4) | 1.000 |
| Amiodarone | 18 (6.2) | 5 (2.0) | 4 (6.8) | 0.047 | 7 (5.0) | 8 (3.1) | 0.481 |
| Meglitinide^a^ | 16 (5.5) | 4 (1.6) | 2 (3.4) | 0.047 | 2 (1.4) | 21 (8.1) | 0.006 |
| **Cancer Therapy** |  |  |  |  |  |  |  |
| Prostatectomy | 40 (13.8) | 43 (17.6) | 10 (16.9) | 0.482 | 21 (15.1) | 39 (15.0) | 1.000 |
| Orchiectomy^a^ | 2 (0.7) | 1 (0.4) | 0 (0.0) | 1.000 | 1 (0.7) | 1 (0.4) | 1.000 |
| Radiation | 60 (20.8) | 58 (23.6) | 9 (15.3) | 0.352 | 30 (21.6) | 54 (20.7) | 0.936 |
| Androgen deprivation therapy |  |  |  |  |  |  |  |
| GnRH agonist^b^ | 116 (40.1) | 110 (44.9) | 22 (37.3) | 0.409 | 57 (41.0) | 116 (44.6) | 0.557 |
| Androgen synthetase inhibitor^c^ | 8 (2.8) | 6 (2.4) | 4 (6.8) | 0.205 | 4 (2.9) | 10 (3.8) | 0.778^a^ |
| Androgen receptor blocker^d^ | 88 (30.4) | 80 (32.7) | 22 (37.3) | 0.570 | 36 (25.9) | 91 (35.0) | 0.081 |

Median (interquartile range) or n (%). P-values were calculated by Wilcoxon rank-sum test, Kruskal-Wallis’s test, or Chi-square test unless marked by superscript letter. Patients were stratified by the average values of serum low-density lipoprotein cholesterol and hemoglobin A1C before the cancer diagnosis.

a. P-values were calculated by Fisher’s exact test.

b. GnRH agonist indicates goserelin, leuprorelin, triptorelin.

c. Androgen synthetase inhibitor indicates abiraterone.

d. Androgen receptor blocker indicates flutamide, bicalutamide, enzalutamide.

CKD-EPI = Chronic Kidney Disease Epidemiology Collaboration; eGFR = estimated glomerular filtration rate; GnRH= Gonadotropin hormone-releasing hormone; HbA1C = hemoglobin A1C; LDL = low-density lipoprotein cholesterol.

**Supplemental Table 4. The association between low-density lipoprotein cholesterol and major cardiovascular event in breast and prostate cancer**

| **Breast Cancer** | | | | | | | | | |
| --- | --- | --- | --- | --- | --- | --- | --- | --- | --- |
| **TMU CRD** | | | | | | | | | |
| LDL | Event/N | Crude Model | *p*-value | | Adjusted Model 1**^a^** | *p*-value | | Adjusted Model 2 **^b^** | *p*-value |
| 100-129  <100  ≥130 | 27/350 | Ref |  | | Ref |  | | Ref |  |
|  | 17/211 | 1.42 [0.76, 2.66] | 0.27 | | 1.13 [0.60, 2.12] | 0.72 | | 0.96 [0.50, 1.84] | 0.89 |
|  | 10/188 | 1.23 [0.58, 2.60] | 0.59 | | 1.41 [0.67, 3.00] | 0.37 | | 1.65 [0.76, 3.55] | 0.21 |
| **TriNetX^g^** | | | | | | | | | |
| LDL | Event/N | Crude Model | *p*-value | | PSM Model 1 **^c^** | *p*-value | | PSM Model 2 **^d^** | *p*-value |
| 100-129  <100  ≥130 | 3,199/74,057 | Ref |  | | Ref |  | | Ref |  |
|  | 4,041/78,045 | 1.26 [1.20, 1.32] | <0.001 | | 1.13 [1.07, 1.18] | <0.001 | | 1.04 [0.99, 1.09] | 0.12 |
|  | 2,476/59,796 | 0.96 [0.91, 1.02] | 0.16 | | 1.01 [0.96, 1.07] | 0.70 | | 1.05 [0.99, 1.11] | 0.08 |
| **Prostate Cancer** | | | | | | | | | |
| **TMU CRD** | | | | | | | | | |
| LDL | Event/N | Crude Model | | *p*-value | Adjusted Model 1 **^a^** | *p*-value | Adjusted Model 2 **^e^** | | P |
| 100-129  <100  ≥130 | 23/200 | Ref | |  | Ref |  | Ref | |  |
|  | 22/204 | 0.98 [0.52, 1.82] | | 0.94 | 0.90 [0.48, 1.68] | 0.74 | 0.82 [0.43, 1.56] | | 0.55 |
|  | 6/54 | 1.44 [0.66, 3.15] | | 0.36 | 1.62 [0.74, 3.57] | 0.23 | 1.58 [0.70, 3.54] | | 0.27 |
| **TriNetX^g^** | | | | | | | | | |
| LDL | Event/N | Crude Model | | *p*-value | PSM Model 1 **^c^** | *p*-value | PSM Model 2 **^f^** | | *p*-value |
| 100-129  <100  ≥130 | 2,757/50,704 | Ref | |  | Ref |  | Ref | |  |
|  | 4,812/74,970 | 1.27 [1.21, 1.33] | | <0.001 | 1.14 [1.08, 1.20] | <0.001 | 0.99 [0.94, 1.05] | | 0.81 |
|  | 1,655/31,711 | 0.96 [0.91, 1.02] | | 0.24 | 1.04 [0.97, 1.11] | 0.27 | 1.09 [1.01, 1.16] | | 0.02 |

The associations were expressed as hazard ratio with 95% confidence intervals.

**^a^**Adjusted Model 1 were estimated using time-dependent cox model adjusted for age.

**^b^**Adjusted Model 2 were estimated using time-dependent cox model adjusted for age, coronary artery disease, peripheral arterial disease, hypertension, diabetes mellitus, hyperlipidemia, atrial fibrillation, valvular heart disease, chronic lung disease, chronic kidney disease, surgery, year of cancer diagnosis, primary cancer site, anthracyclines, anti-HER2 Therapy, hormone therapy.

**^c^**PSM Model 1 were estimated using cox model and matched by propensity score for age.

**^d^**PSM Model 2 were estimated using cox model and matched by propensity score for age, coronary artery disease, peripheral arterial disease, hypertension, diabetes mellitus, hyperlipidemia, atrial fibrillation, valvular heart disease, chronic lung disease, chronic kidney disease, anti-HER2 therapy.

**^e^**Adjusted Model 2 were estimated using time-dependent cox model adjusted for age, coronary artery disease, peripheral arterial disease, hypertension, diabetes mellitus, hyperlipidemia, atrial fibrillation, valvular heart disease, chronic lung disease, chronic kidney disease, surgery, year of cancer diagnosis, primary cancer site, GnRH agonist, androgen synthesis inhibitor, androgen receptor blocker.

**^f^**PSM Model 2 were estimated using cox model and matched by propensity score for age, coronary artery disease, peripheral arterial disease, hypertension, diabetes mellitus, hyperlipidemia, atrial fibrillation, valvular heart disease, chronic lung disease, chronic kidney disease, GnRH agonist, androgen synthesis inhibitor, androgen receptor blocker.

**^g^** p-value for log-rank test.

LDL = low-density lipoprotein cholesterol; PSM = Propensity score matching; TMU CRD = Taipei Medical University Clinical Research Database; Ref = reference.

**Supplemental Table 5. The association between hemoglobin A1C and major cardiovascular event in breast and prostate cancer**

| **Breast Cancer** | | | | | | | | | |
| --- | --- | --- | --- | --- | --- | --- | --- | --- | --- |
| **TMU CRD** | | | | | | | | | |
| HbA1C | Event/N | Crude Model | *p*-value | | Adjusted Model 1**^a^** | *p*-value | | Adjusted Model 2 **^b^** | *p*-value |
| <6 | 6/153 | Ref |  | | Ref |  | | Ref |  |
| ≥6 | 33/322 | 1.78 [0.78, 4.06] | 0.17 | | 1.70 [0.74, 3.87] | 0.21 | | 1.42 [0.57, 3.57] | 0.46 |
| **TriNetX^g^** | | | | | | | | | |
| HbA1C | Event/N | Crude Model | *p*-value | | PSM Model 1 **^c^** | *p*-value | | PSM Model 2 **^d^** | *p*-value |
| <6 | 2,728/71,161 | Ref |  | | Ref |  | | Ref |  |
| ≥6 | 3,063/48,418 | 1.74 [1.66, 1.84] | <0.001 | | 1.46 [1.38, 1.54] | <0.001 | | 1.18 [1.110, 1.26] | <0.001 |
| **Prostate Cancer** | | | | | | | | | |
| **TMU CRD** | | | | | | | | | |
| HbA1C | Event/N | Crude Model | | *p*-value | Adjusted Model 1 **^a^** | *p*-value | Adjusted Model 2 **^e^** | | P |
| <6 | 5/101 | Ref | |  | Ref |  | Ref | |  |
| ≥6 | 22/216 | 1.48 [0.59, 3.68] | | 0.40 | 1.45 [0.58, 3.61] | 0.43 | 1.60 [0.58, 4.44] | | 0.36 |
| **TriNetX^g^** | | | | | | | | | |
| HbA1C | Event/N | Crude Model | | *p*-value | PSM Model 1 **^c^** | *p*-value | PSM Model 2 **^f^** | | *p*-value |
| <6 | 2,811/51,750 | Ref | |  | Ref |  | Ref | |  |
| ≥6 | 3,221/43,029 | 1.48 [1.41, 1.56] | | <0.001 | 1.37 [1.30, 1.45] | <0.001 | 1.16 [1.09, 1.23] | | <0.001 |

The associations were expressed as hazard ratio with 95% confidence intervals.

**^a^**Adjusted Model 1 were estimated using time-dependent cox model adjusted for age.

**^b^**Adjusted Model 2 were estimated using time-dependent cox model adjusted for age, coronary artery disease, peripheral arterial disease, hypertension, diabetes mellitus, hyperlipidemia, atrial fibrillation, valvular heart disease, chronic lung disease, chronic kidney disease, surgery, year of cancer diagnosis, primary cancer site, anthracyclines, anti-HER2 Therapy, hormone therapy.

**^c^**PSM Model 1 were estimated using cox model and matched by propensity score for age.

**^d^**PSM Model 2 were estimated using cox model and matched by propensity score for age, coronary artery disease, peripheral arterial disease, hypertension, diabetes mellitus, hyperlipidemia, atrial fibrillation, valvular heart disease, chronic lung disease, chronic kidney disease, anti-HER2 therapy.

**^e^**Adjusted Model 2 were estimated using time-dependent cox model adjusted for age, coronary artery disease, peripheral arterial disease, hypertension, diabetes mellitus, hyperlipidemia, atrial fibrillation, valvular heart disease, chronic lung disease, chronic kidney disease, surgery, year of cancer diagnosis, primary cancer site, GnRH agonist, androgen synthesis inhibitor, androgen receptor blocker.

**^f^**PSM Model 2 were estimated using cox model and matched by propensity score for age, coronary artery disease, peripheral arterial disease, hypertension, diabetes mellitus, hyperlipidemia, atrial fibrillation, valvular heart disease, chronic lung disease, chronic kidney disease, GnRH agonist, androgen synthesis inhibitor, androgen receptor blocker.

**^g^** p-value for log-rank test.

HbA1C = hemoglobin A1C; PSM = Propensity score matching; TMU CRD = Taipei Medical University Clinical Research Database; Ref = reference.

**Supplemental Table 6. The association between hemoglobin A1C and total death or cancer recurrence in breast and prostate cancer**

| **Breast Cancer** | | | | | | | | | |
| --- | --- | --- | --- | --- | --- | --- | --- | --- | --- |
| **TMU CRD** | | | | | | | | | |
| HbA1C | Event/N | Crude Model | *p*-value | | Adjusted Model 1**^a^** | *p*-value | | Adjusted Model 2 **^b^** | *p*-value |
| <6 | 25/169 | Ref |  | | Ref |  | | Ref |  |
| ≥6 | 71/371 | 1.06 [0.67, 1.67] | 0.80 | | 0.96 [0.61, 1.51] | 0.86 | | 0.90 [0.54, 1.51] | 0.68 |
| **TriNetX^g^** | | | | | | | | | |
| HbA1C | Event/N | Crude Model | *p*-value | | PSM Model 1 **^c^** | *p*-value | | PSM Model 2 **^d^** | *p*-value |
| <6 | 12,565/91,647 | Ref |  | | Ref |  | | Ref |  |
| ≥6 | 13,005/68,944 | 1.43 [1.40, 1.47] | <0.001 | | 1.31 [1.27, 1.34] | <0.001 | | 1.15 [1.12, 1.19] | <0.001 |
| **Prostate Cancer** | | | | | | | | | |
| **TMU CRD** | | | | | | | | | |
| HbA1C | Event/N | Crude Model | | *p*-value | Adjusted Model 1 **^a^** | *p*-value | Adjusted Model 2 **^e^** | | P |
| <6 | 19/139 | Ref | |  | Ref |  | Ref | |  |
| ≥6 | 63/260 | 1.18 [0.73, 1.91] | | 0.49 | 1.21 [0.75, 1.95] | 0.44 | 1.25 [0.74, 2.12] | | 0.41 |
| **TriNetX^g^** | | | | | | | | | |
| HbA1C | Event/N | Crude Model | | *p*-value | PSM Model 1 **^c^** | *p*-value | PSM Model 2 **^f^** | | *p*-value |
| <6 | 13,178/76,954 | Ref | |  | Ref |  | Ref | |  |
| ≥6 | 15,080/68,333 | 1.37 [1.34, 1.40] | | <0.001 | 1.30 [1.27, 1.33] | <0.001 | 1.14 [1.11, 1.17] | | <0.001 |

The association were expressed as hazard ratio with 95% confidence intervals.

**^a^**Adjusted Model 1 were estimated using time-dependent cox model adjusted for age.

**^b^**Adjusted Model 2 were estimated using time-dependent cox model adjusted for age, coronary artery disease, peripheral arterial disease, hypertension, diabetes mellitus, hyperlipidemia, atrial fibrillation, valvular heart disease, chronic lung disease, chronic kidney disease, surgery, year of cancer diagnosis, primary cancer site, anthracyclines, anti-HER2 Therapy, hormone therapy.

**^c^**PSM Model 1 were estimated using cox model and matched by propensity score for age.

**^d^**PSM Model 2 were estimated using cox model and matched by propensity score for age, coronary artery disease, peripheral arterial disease, hypertension, diabetes mellitus, hyperlipidemia, atrial fibrillation, valvular heart disease, chronic lung disease, chronic kidney disease, anti-HER2 therapy.

**^e^**Adjusted Model 2 were estimated using time-dependent cox model adjusted for age, coronary artery disease, peripheral arterial disease, hypertension, diabetes mellitus, hyperlipidemia, atrial fibrillation, valvular heart disease, chronic lung disease, chronic kidney disease, surgery, year of cancer diagnosis, primary cancer site, GnRH agonist, androgen synthesis inhibitor, androgen receptor blocker.

**^f^**PSM Model 2 were estimated using cox model and matched by propensity score for age, coronary artery disease, peripheral arterial disease, hypertension, diabetes mellitus, hyperlipidemia, atrial fibrillation, valvular heart disease, chronic lung disease, chronic kidney disease, GnRH agonist, androgen synthesis inhibitor, androgen receptor blocker.

**^g^** Information of cancer recurrence was unavailable. p-value for log-rank test.

HbA1C = hemoglobin A1C; PSM = Propensity score matching; TMU CRD = Taipei Medical University Clinical Research Database; Ref = reference.

**Supplemental Figure 1.** The diagram of the cohort selection for breast cancer in the Taipei Medical University Clinical Research Database. HbA1C = hemoglobulin A1C; LDL = low-density protein cholesterol; TMUCRD = Taipei Medical University Clinical Research Database.


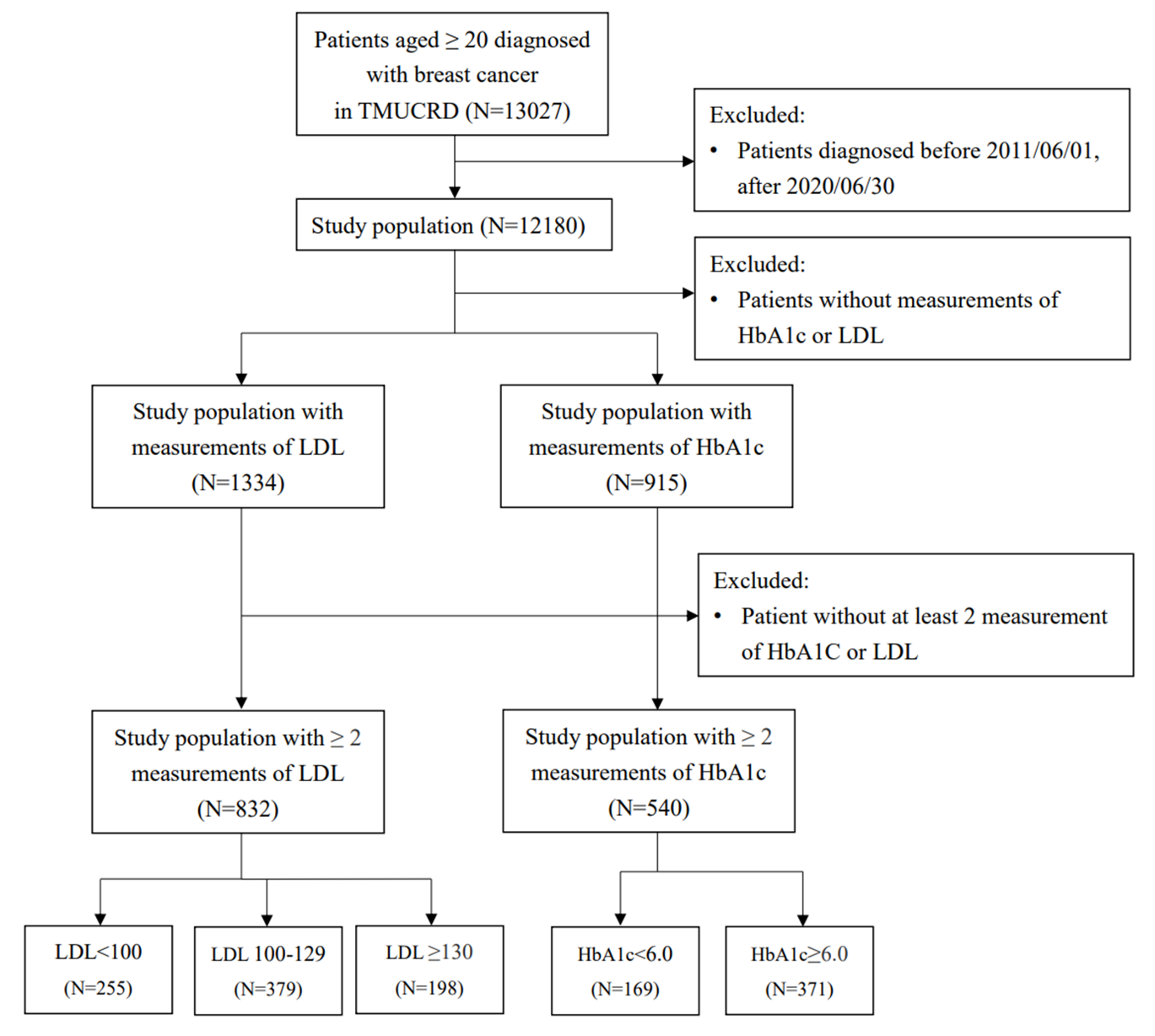


**Supplemental Figure 2.** The diagram of the cohort selection for prostate cancer in the Taipei Medical University Clinical Research Database. HbA1C = hemoglobulin A1C; LDL = low-density protein cholesterol; TMUCRD = Taipei Medical University Clinical Research Database.


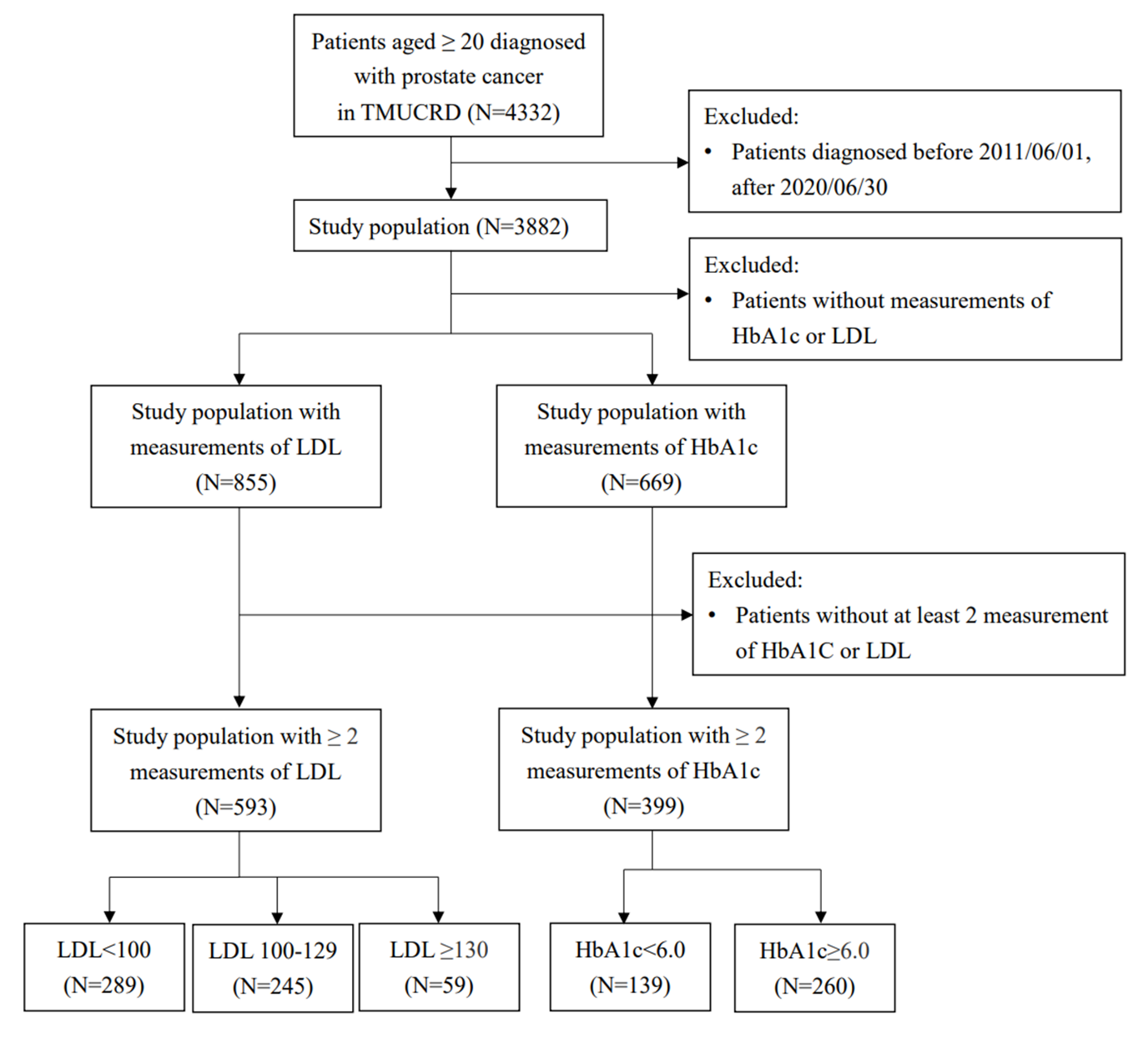


**Supplemental Figure 3. The diagram of the cohort selection for breast cancer in the TriNetX research network.**


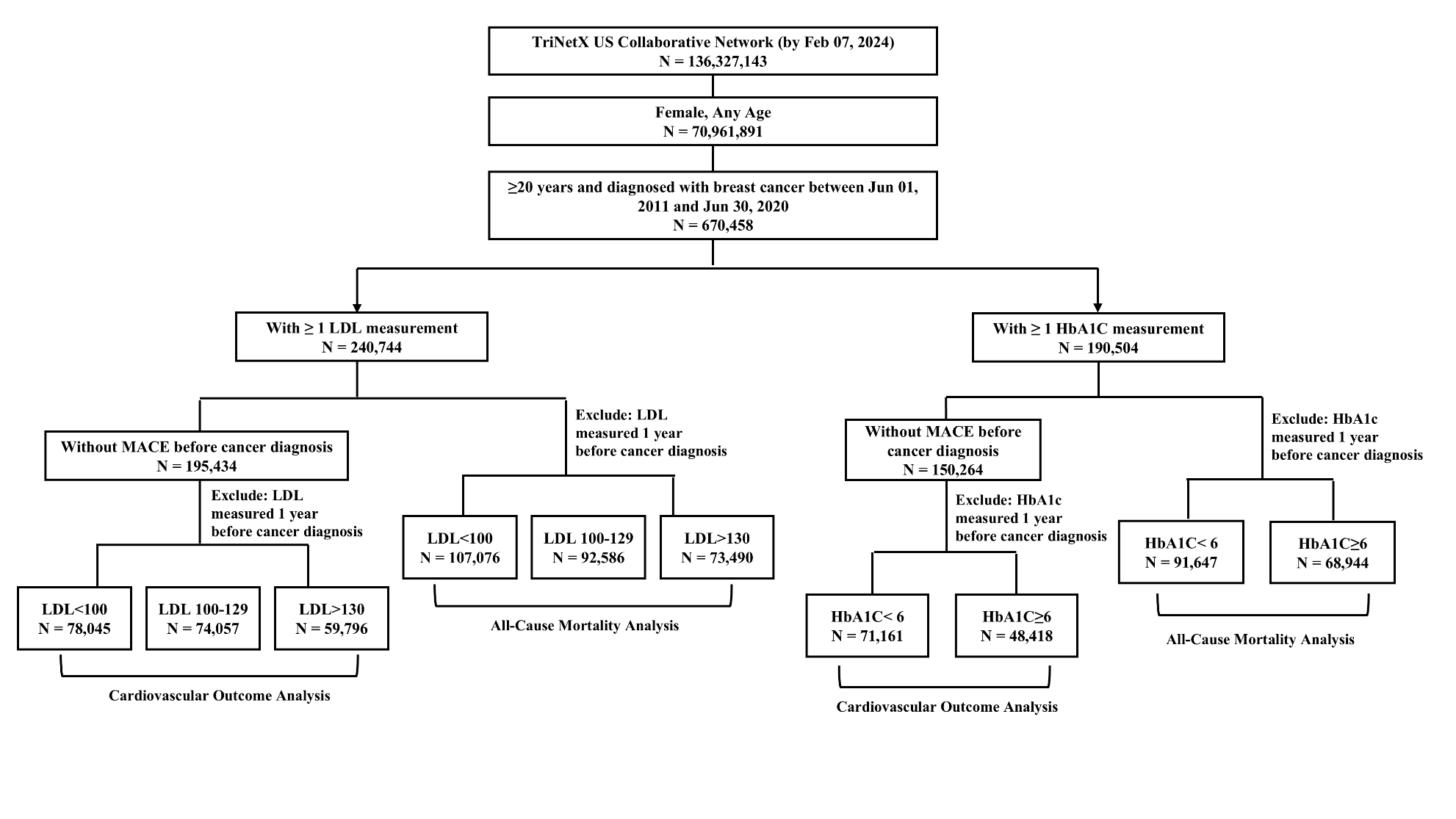


The built-in functions of the TriNetX network limited the ability to customize longitudinal data collection, potentially leading to repeated patient selection. The propensity score matching method was applied to address confounding by indication of testing LDL or HbA1C. HbA1C = hemoglobulin A1C; LDL = low-density protein cholesterol.

**Supplemental Figure 4. The diagram of the cohort selection for prostate cancer in the TriNetX research network.**


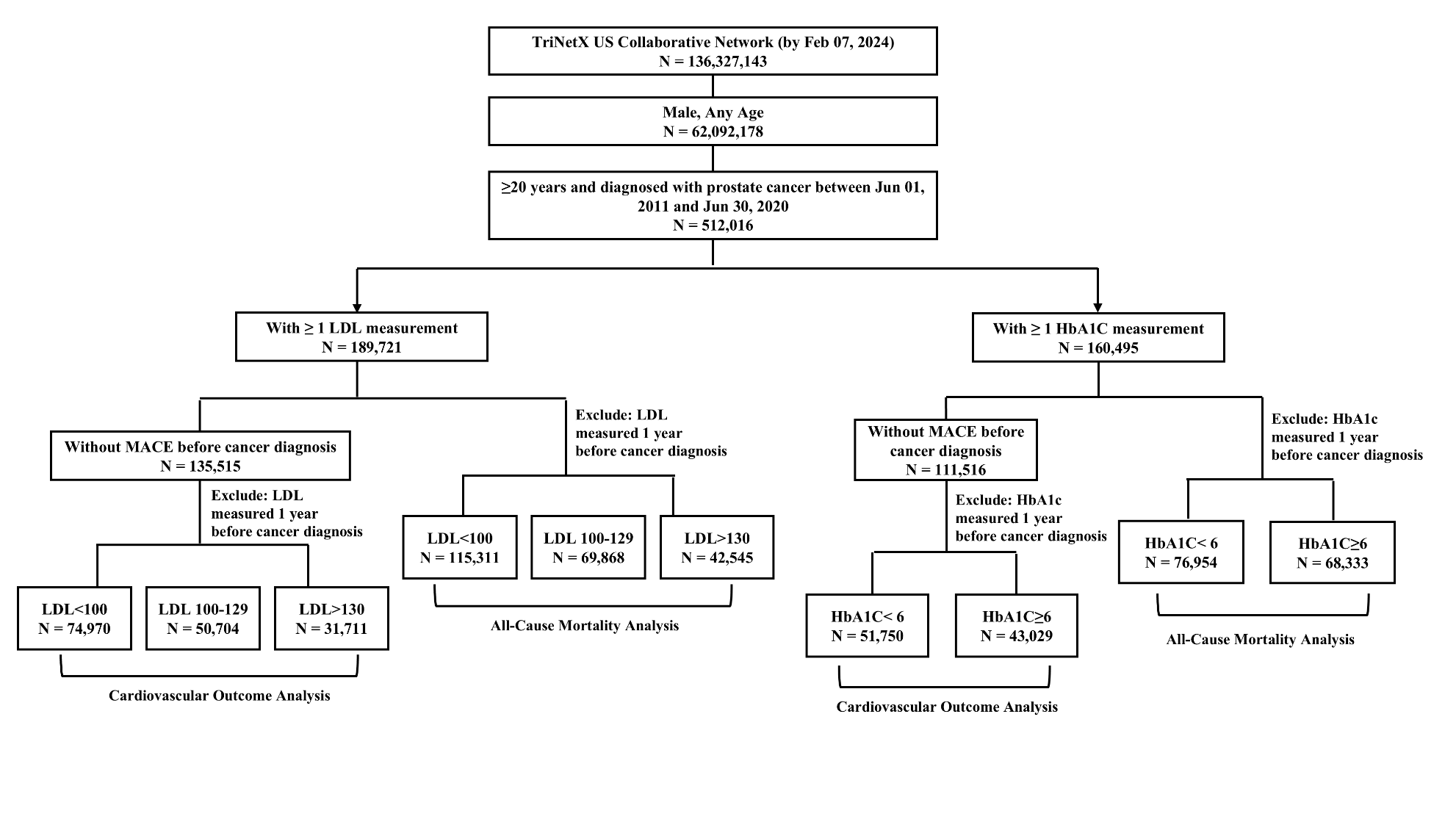


The built-in functions of the TriNetX network limited the ability to customize longitudinal data collection, potentially leading to repeated patient selection. The propensity score matching method was applied to address confounding by indication of testing LDL or HbA1C. HbA1C = hemoglobulin A1C; LDL = low-density protein cholesterol.
